# Supplementary material for: Three-dimensional magnetic nanotextures with high-order vorticity in soft magnetic wireframes
Source: Nat Commun. 2024 Mar 11;15:2193. doi: 10.1038/s41467-024-46403-8 (PMC10928081; doi:10.1038/s41467-024-46403-8)
Supplement: Supplementary file 1 — supplementary information [file 41467_2024_46403_MOESM1_ESM.pdf]

Supplementary Information to
"Three dimensional magnetic nanotextures with high-order vorticity in soft magnetic wireframes"

Oleksii M. Volkov,[1,*] Oleksandr V. Pylypovskyi,[1,2,†] Fabrizio Porrati,[3,‡] Florian Kronast,[4] Jose A. Fernandez-Roldan,[1] Attila Kákay,[1] Alexander Kuprava,[3] Sven Barth,[3] Filipp N. Rybakov,[5] Olle Eriksson,[5,6] Sebastian Lamb-Camarena,[7,8] Pavlo Makushko,[1] Mohamad-Assaad Mawass,[9] Shahrukh Shakeel,[1] Oleksandr V. Dobrovolskiy,[7] Michael Huth,[3] and Denys Makarov[1,§]

[1] *Helmholtz-Zentrum Dresden-Rossendorf e.V., Institute of Ion Beam Physics and Materials Research, Bautzner Landstr. 400, 01328 Dresden, Germany*
[2] *Kyiv Academic University, 03142 Kyiv, Ukraine*
[3] *Physikalisches Institut, Johann Wolfgang Goethe-Universität Frankfurt am Main, Max-von-Laue-Str. 1, 60438 Frankfurt am Main, Germany*
[4] *Helmholtz-Zentrum Berlin für Materialien und Energie, Albert-Einstein-Str. 15, 12489 Berlin, Germany*
[5] *Department of Physics and Astronomy, Uppsala University, Box-516, Uppsala SE-751 20, Sweden*
[6] *Wallenberg Initiative Materials Science for Sustainability, Uppsala University, 75121 Uppsala, Sweden*
[7] *University of Vienna, Faculty of Physics, Nanomagnetism and Magnonics, Superconductivity and Spintronics Laboratory, Währinger Str. 17, 1090 Vienna, Austria*
[8] *University of Vienna, Vienna Doctoral School in Physics, Boltzmanngasse 5, A-1090 Vienna, Austria*
[9] *Helmholtz-Zentrum Berlin für Materialien und Energie, Albert-Einstein-Str. 15, 12489 Berlin, Germany*
Present address: Department of Interface Science, Fritz-Haber-Institut der Max-Planck-Gesellschaft, Faradayweg 4 – 6, 14195, Berlin, Germany

Contents

1. Transformation between bulk and surface antivortices 3

2. Impact of the tetrapod geometry on magnetic textures 4
   A. Rotation angle between top and bottom tetrapod segments 4
   B. Opening angle 4
   C. Length of the tetrapod line segments 4
   D. Radius of line segments 6
   E. Another example of tetrapods with antivortices: lattice model 7

3. Experimental tetrapod structures 8

4. Complex wireframe structures 9

5. XMCD-PEEM characterization 11

6. Magnetic hysteresis of a tetrapod structure 13

7. Magnetic wireframes: examples 15
   A. Six-arm star geometry 15
   B. Tripod 15
   C. Pentapod 16
   D. Pyramid 17
   E. Cube 18

* o.volkov@hzdr.de
† o.pylypovskyi@hzdr.de
‡ porrati@physik.uni-frankfurt.de
§ d.makarov@hzdr.de

8. Stray fields in wireframe structures 20
A. Symmetry of stray field isosurfaces in homeomorphic tetrapods 20
B. Classification of stray field topology 21
C. Inhomogeneous stray fields: Field orientability for applications 21
D. Geometrically-induced robustness of the stray field of tetrapods exposed to an external magnetic field 21

9. Supplemetary Videos 22

References 22

Supplementary Section 1. Transformation between bulk and surface antivortices

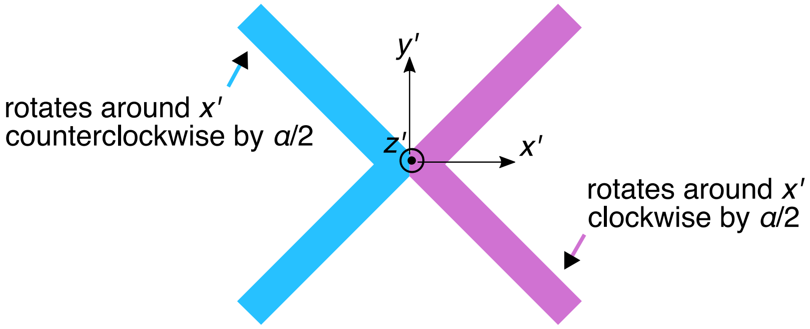

Supplementary Figure 1. Schematic image of the tetrapod geometry with two parts rotated relative to each other.

We consider the transition between the bulk and surface states in a tetrapod by varying the rotation angle α between the top and bottom parts of the tetrapod. We propose a continuous field configuration $\mathbf{m}(x', y', z')$ that represents the bulk vortex state for $\alpha = 0$ (planar tetrapod in the $x'y'$ plane) and the surface vortex state for $\alpha = \pi$ (planar tetrapod, but in the $x'z'$ plane). Let the radius of each tetrapod line segment be equal to r. The model transformation for the antivortex solution in the central region of the tetrapod can be derived by the action of the rotation matrix $\mathbf{M}$ on a trivial collinear state, namely:

$$\mathbf{m} = \mathbf{M} \cdot \begin{pmatrix} 0 \\ 0 \\ -1 \end{pmatrix}, \tag{S1}$$

with the expression for the objective matrix $\mathbf{M}$,

$$\mathbf{M} = \mathbf{R}(\vartheta_1, \mathbf{k}_1) \cdot \mathbf{R}(\vartheta_2, \mathbf{k}_2), \tag{S2}$$

where $\mathbf{R}(\vartheta, \mathbf{k})$ is the rotation transformation matrix that denotes rotation by the angle ϑ around a given vector $\boldsymbol{k} = \{k_x, k_y, k_z\}$ (the Rodrigues' rotation matrix)

$$\mathbf{R}(\vartheta, \mathbf{k}) = \mathbf{I} + \sin(\vartheta)\mathbf{K} + (1 - \cos(\vartheta))\mathbf{K} \cdot \mathbf{K}, \qquad \mathbf{I} \equiv \begin{pmatrix} 1 & 0 & 0 \\ 0 & 1 & 0 \\ 0 & 0 & 1 \end{pmatrix}, \qquad \mathbf{K} \equiv \begin{pmatrix} 0 & -k_z & k_y \\ k_z & 0 & -k_x \\ -k_y & k_x & 0 \end{pmatrix}, \tag{S3}$$

with $\mathbf{I}$ being the identity matrix and ϑ_1 and ϑ_2 being rotation angles

$$\vartheta_1 = \left(1 - \frac{\alpha}{\pi}\right) \cdot \frac{\pi}{2} \cdot \min\left(\frac{\rho}{r}, 1\right), \quad \vartheta_2 = \frac{\alpha}{\pi} \cdot \frac{\pi}{4} \cdot \max\left(-1, \min\left(\frac{x'}{r}, 1\right)\right) \cdot \max\left(-1, \min\left(\frac{z'}{r}, 1\right)\right), \tag{S4}$$

around the following vectors

$$\mathbf{k}_1 = \begin{pmatrix} \cos(-\varphi) \\ \sin(-\varphi) \\ 0 \end{pmatrix}, \quad \mathbf{k}_2 = \begin{pmatrix} 0 \\ 1 \\ 0 \end{pmatrix}, \tag{S5}$$

which are set up for the cylindrical coordinate system $\{\rho, \varphi, z'\}$: $\rho \equiv \sqrt{x'^2 + y'^2}$ and $\varphi \equiv \arg(x' + i\,y')$. Thus, expression (S2) is a composition of two rotation matrices, each of which is intermediate between the trivial one and another rotation matrix corresponding to either the bulk vortex state or the surface vortex state. The resulting transformation dynamic is shown in the Supplementary Video 1.

Supplementary Section 2. Impact of the tetrapod geometry on magnetic textures

To investigate the influence of geometric characteristics of a tetrapod on the resulting equilibrium magnetic states, we performed simulations varying geometric parameters including the rotation angle, opening angle, line segment length and its diameter.

A. Rotation angle between top and bottom tetrapod segments

We define α as the rotation angle between the top and bottom parts of the tetrapod. Here, we consider values of α from $0°$ to $180°$ with a step of $10°$. For each case, the geometry is constructed from rounded 1.3 μm long straight nanowire segments with a radius of 57 nm and opening angle of $90°$ between linear segments of its top and bottom parts. Examples of tetrapod geometries which are used in micromagnetic simulations are shown in Supplementary Fig. 2. For instance, the tetrapod with the rotation angle $\alpha = 0°$ stabilizes at equilibrium a bulk antivortex state with a Bloch line going through the connection area of the four line segments. The surface antivortex state is a metastable state for all geometries but $\alpha = 90°$, where it possesses the same energy as the bulk one. The resulting total vorticity $Q^{\Sigma} = +2$ for all cases.

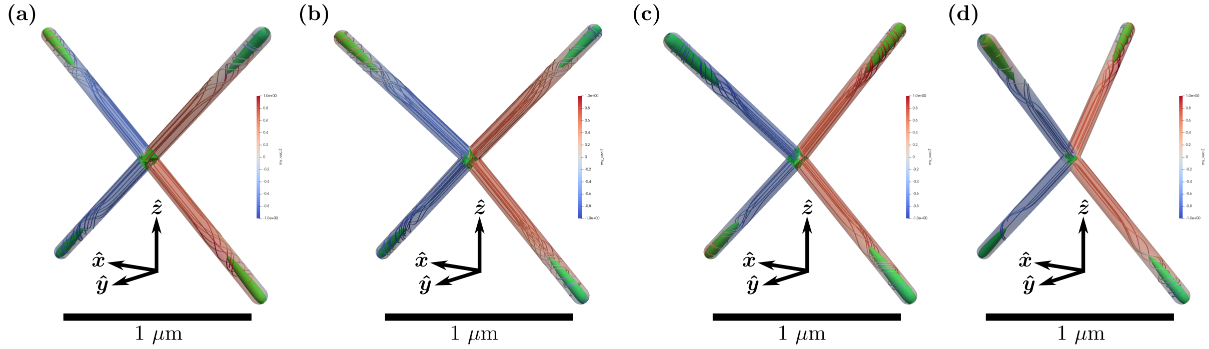

Supplementary Figure 2. **The tetrapod geometry with bulk and surface antivortex states.** Panels show the equilibrium magnetic state of tetrapods with different azimuthal rotation angles α of $0°$, $30°$, $60°$ and $90°$ between the planes accommodating the top and bottom parts of the tetrapod. Green regions in each geometry show the topological charge density distribution.

B. Opening angle

The opening angle β is defined as the angle between the bottom segments of the tetrapod; the angle between the top segments of the tetrapod is kept the same. We perform micromagnetic simulations for the tetrapod geometries with the rotation angle $\alpha = 90°$ and constructed from the 1.3 μm long linear nanowires with the radius of 57 nm and opening angles $\beta \in [30°; 45°; 60°; 75°; 90°]$. Starting from different initial configurations, we stabilize various equilibrium magnetic textures presented in Supplementary Fig. 3. In the case of opening angle $\beta = 30°$, the equilibrium state acquires the total vorticity $Q^{\Sigma} = +2$, with four vortices and two antivortices. An additional shape anisotropy that appears in the central tetrapod region, due to the spatial elongation, leads to the formation of a hybrid vortex-antivortex state with two antivortices at the surface being positioned along the $\hat{\boldsymbol{z}}$-axis, and a bulk vortex Bloch line inside the central region. For geometries with the opening angle β larger than $45°$, both antivortex states are positioned in the $\hat{\boldsymbol{x}}\hat{\boldsymbol{y}}$ plane of the central tetrapod region.

C. Length of the tetrapod line segments

To investigate the influence of shortening of the tetrapod line segments on the equilibrium magnetic states, we perform micromagnetic simulations for geometries constructed from rounded linear nanowires of different length, $L \in [300; 500; 700; 900; 1100; 1300]$ nm, with the same radius $r = 57$ nm, the rotation angle $\alpha = 90°$ and opening angle $\beta = 90°$. In the case of the tetrapod with the shortest line segments ($L = 300$ nm), the resulting equilibrium magnetic state possesses an additional vortex-antivortex pair in the central region due to the formation of the bulk vortex Bloch lines that intertwine in the connection area, see Supplementary Fig. 4(a). Thus, the surface magnetization

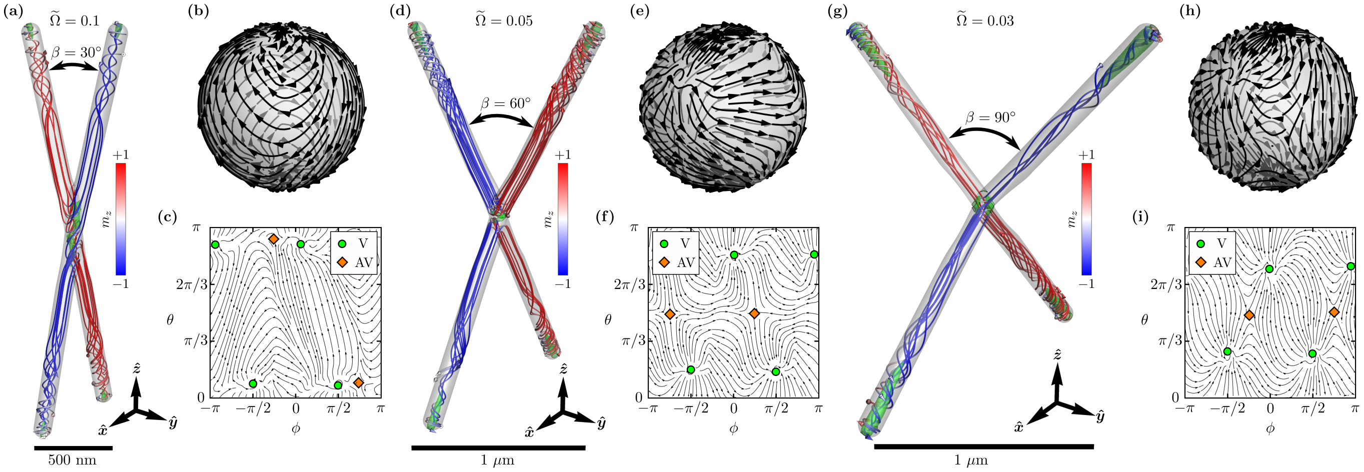

Supplementary Figure 3. **Effect of the opening angle on magnetic states in tetrapods. (a)** The tetrapod geometry with an opening angle $\beta = 30°$ leads to the effective elongation of the central area along the $\hat{\boldsymbol{z}}$-axis. Green regions correspond to the isosurfaces in the distribution of the topological charge density $\widetilde{\Omega} = 0.1$. Maxima in these distributions correspond to the locations of topologically non-trivial textures. Stream-arrows depict the magnetization state inside the tetrapod. **(b)** Mapping of the surface magnetization distribution of the tetrapod onto a unit sphere. **(c)** Angular projection of the sphere in azimuthal and polar coordinates, (θ, ϕ). Panels **(d − f)** and **(g − i)** show the corresponding information for the tetrapods with the opening angle $\beta = 60°$ and $\beta = 90°$, respectively. The length of the line segments forming the here studied tetrapod geometries is $L = 1.3$ μm, the radius of line segments is $r = 57$ nm, rotation angle $\alpha = 90°$.

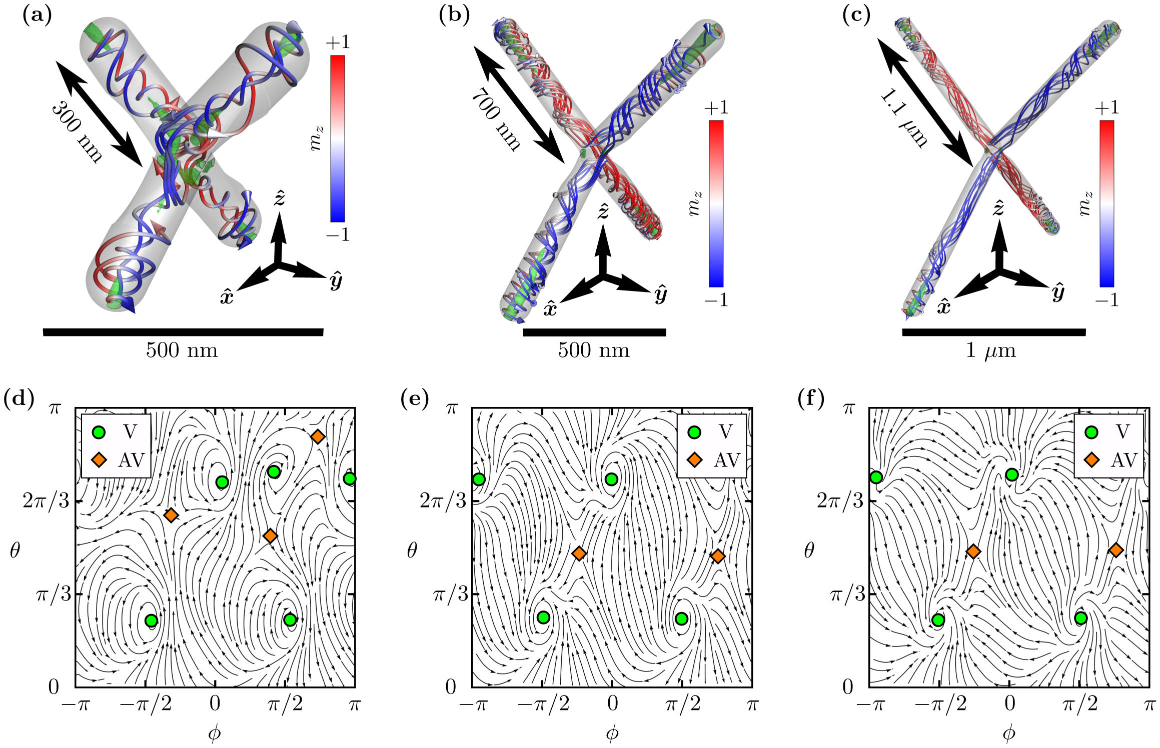

Supplementary Figure 4. **Effect of the line segment length of the tetrapod geometry on the magnetic texture. (a − c)** Equilibrium magnetic states of tetrapods constructed from 300 nm, 700 nm and 1.1 μm long line segments. Green regions inside each geometry represent the isosurface of the topological charge density distribution, that reach their maxima near topologically non-trivial textures. Arrows show magnetization stream-lines of the magnetic states. **(d − f)** Angular projections of the surface magnetization distribution shown in panels **(a − c)**, respectively, on an elemental sphere. Here, (θ, ϕ) are azimuthal and polar coordinates. Green circles indicate the position of vortices and orange diamonds show the position of antivortices. The radius of the line segments forming the tetrapod geometries studied here is 58 nm, the rotation angle $\alpha = 90°$ and the opening angle $\beta = 90°$.

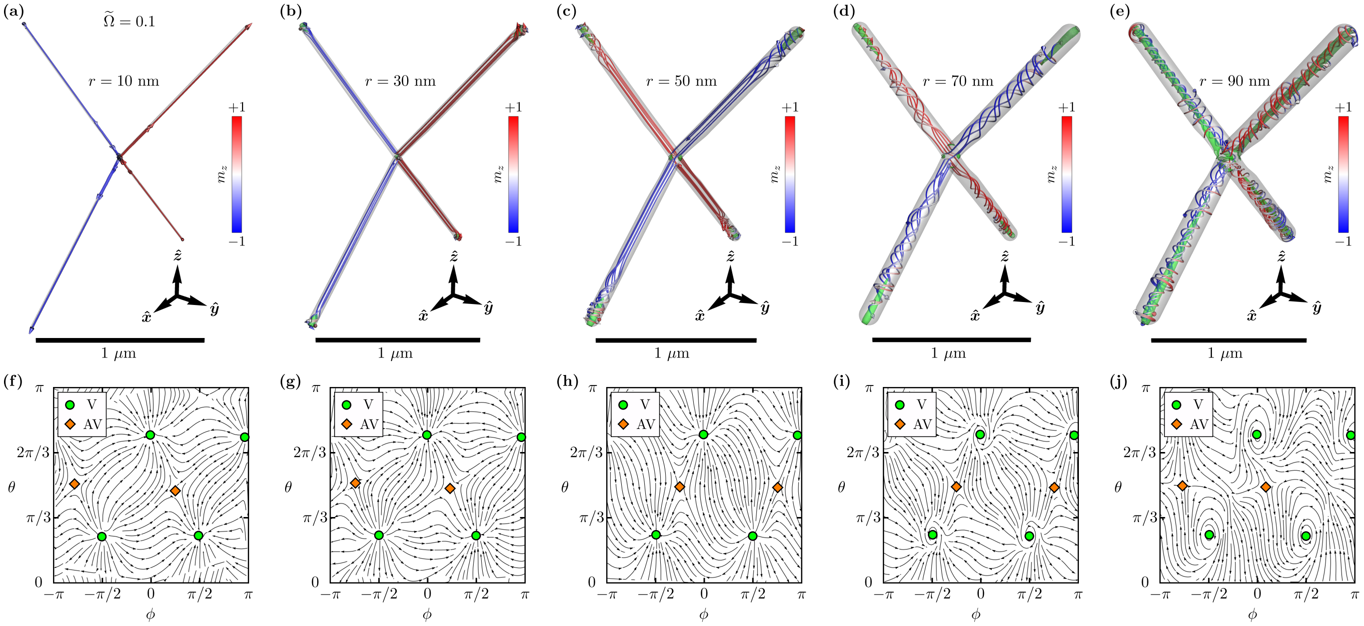

Supplementary Figure 5. **Effect of the radius of line segments forming the tetrapod geometry on magnetic textures.** $(\mathbf{a}-\mathbf{e})$ Equilibrium magnetization states of the tetrapods with $r = [10; 30; 50; 70; 90]$ nm, respectively. Green regions correspond to the isosurfaces in the distribution of the topological charge density $\widetilde{\Omega} = 0.1$. Maxima in these distributions correspond to the locations of topologically non-trivial textures. Stream-arrows depict the magnetization state inside the tetrapod. $(\mathbf{f}-\mathbf{j})$ Angular projections of the surface magnetization distributions shown in panels $(\mathbf{a}-\mathbf{e})$, respectively. Green circles indicate the position of vortices and orange diamonds show the position of antivortices. The length of the line segments forming the here studied tetrapod geometries is $L = 1.3$ μm, the rotation angle $\alpha = 90^\circ$ and opening angle $\beta = 90^\circ$.

distribution acquires 5 vortices and 3 antivortices, resulting in the total vorticity $Q^\Sigma = +2$ being in line with the Euler characteristic of the geometry $\chi = +2$, see Supplementary Fig. 4(c). With the elongation of the line segments to $L = 700$ nm, the vortex structure appears only as a surface state at their ends without the formation of Bloch lines along segments, see Supplementary Fig. 4(b). This results in the emergence of a pair of antivortices at the surface of the connection region despite the magnetization curling in linear segments near the connection area, see Supplementary Fig. 4(e). Further elongation of the line segments to $L = 1.1$ μm does not result in a change of the total number of magnetic textures in the tetrapod, but it leads to the formation of homogeneously magnetized linear segments in the central area of the tetrapod with a reduction of the curling of magnetization at the surface, see Supplementary Fig. 4(c) and (f). We note that the segment length determines the strength of the interaction between vortices at the segment ends and antivortices in the central part of the geometry. If the segments are short enough, the vortex cores can substantially affect antivortices in the center, even leading to the creation of an additional vortex-antivortex pair in equilibrium.

D. Radius of line segments

To study the influence of the radius of line segments forming the tetrapod geometry on the equilibrium magnetic states, we construct tetrapods with $L = 1.3$ μm long line segments, the opening angle $\beta = 90^\circ$ and the rotation angle $\alpha = 90^\circ$, but with different nanowire radii $r \in [10; 20; 30; 40; 50; 60; 70; 80; 90; 100]$ nm. For all constructed geometries, we observe the formation of antivortex states in the central region of the tetrapod at equilibrium, see Supplementary Fig. 5. In the case of the tetrapod constructed from narrow line segments with $r \leq 30$ nm, the line segments are homogeneously magnetized. The topological defect in the magnetic texture is presented by the out-of-surface magnetization in the flower state rather than the classical vortex distribution. Still, this state topologically equivalent to the vortex one as it is shown by its angular projection of the surface magnetization on the elemental sphere, see Fig. 5f. The increase of the radius of the line segments ($40 \leq r \leq 70$ nm) leads to the formation of a surface vortex distribution at their ends. For the case of line segments with $r \geq 80$ nm, the surface vortices fill the entire volume of nanowires with the vortex Bloch line. The formation of the bulk vortex Bloch lines may potentially introduce additional vortex-antivortex surface pairs in the central region of the tetrapod.

E. Another example of tetrapods with antivortices: lattice model

In this section we consider the classical Heisenberg model on a simple cubic lattice:

$$E_{\mathrm{TOT}} = -J\sum_{\langle i,j\rangle}\mathbf{s}_i\cdot\mathbf{s}_j - \frac{1}{2}\frac{\mu_0\mu_{\mathrm{a}}^2}{4\pi}\sum_i \mathbf{s}_i\cdot\left(\sum_{j\neq i}\nabla\times\nabla\times\frac{\mathbf{s}_j}{|\mathbf{r}-\mathbf{r}_j|}\right)_{\mathbf{r}=\mathbf{r}_i}, \tag{S6}$$

where J is the exchange coupling constant and μ_{a} is the magnetic moment per site. We set $J = 0.213$, $\mu_0\mu_{\mathrm{a}}^2 = 57.4$, and $a = 3.06$ c.u., where a is the lattice constant. To create the tetrapod shape, we apply a stencil to a rectangular array of 128×128×128 spins. The stencil includes 4 cylinders with length and radius equal to 222 c.u. and 18 c.u. respectively. The parameters listed above are chosen so that the tetrapod shown in Supplementary Fig. 4(b) approximately meets the micromagnetic limit of the Hamiltonian (S6), including that the exchange length $\ell = 1$ c.u. The key differences between the model (S6) and the micromagnetic one are discreteness and the absence of spatial approximation errors. At the same time, model (S6) takes into account interactions of the same nature as in the micromagnetic model. Solutions were obtained by direct energy minimization. To do this, we use the nonlinear conjugate gradient method implemented in the Excalibur software [**?**].

The Supplementary Video demonstrates the energy minimization process for a tetrapod with a variable rotation angle α. At the beginning, the magnetic configuration corresponds to the bulk antivortex state. Then two halves of the tetrapod rotate relative to each other starting from $\alpha = 0^\circ$ and up to the angle of 180°. Thus, the flat tetrapod transforms into itself geometrically, but the final magnetic configuration corresponds to the state with surface antivortices. The magnetization configuration is demonstrated by surface spins, as well as depicted by the isosurfaces $|\Omega| = \mathrm{const}$.

Supplementary Section 3. Experimental tetrapod structures

In addition to the magnetic tetrapod geometry discussed in the main text, we fabricated tetrapod structures of different geometries by means of focused electron-beam-induced deposition (FEBID) from the same precursor $HCo_3Fe(CO)_{12}$ gas [?], see Supplementary Fig. 6 and 7. The differences between the fabricated Co_3Fe tetrapods are in the opening angles of the top and bottom segments of 95° and 108° (Supplementary Fig. 6), and 113° and 106° (Supplementary Fig. 7), respectively. These geometries are constructed from linear segments with elliptical cross-section due to the different electron dwell time upon horizontal and vertical structure deposition. Namely, the first tetrapod geometry (Supplementary Fig. 6) has an averaged elliptical cross-section of 40 nm semi-minor and 72 nm semi-major axes, while the second one (Supplementary Fig. 7) has 55 nm and 67 nm semi axes. The resulting height of the tetrapods is 1.38 μm (Supplementary Fig. 6) and 1.5 μm (Supplementary Fig. 7).

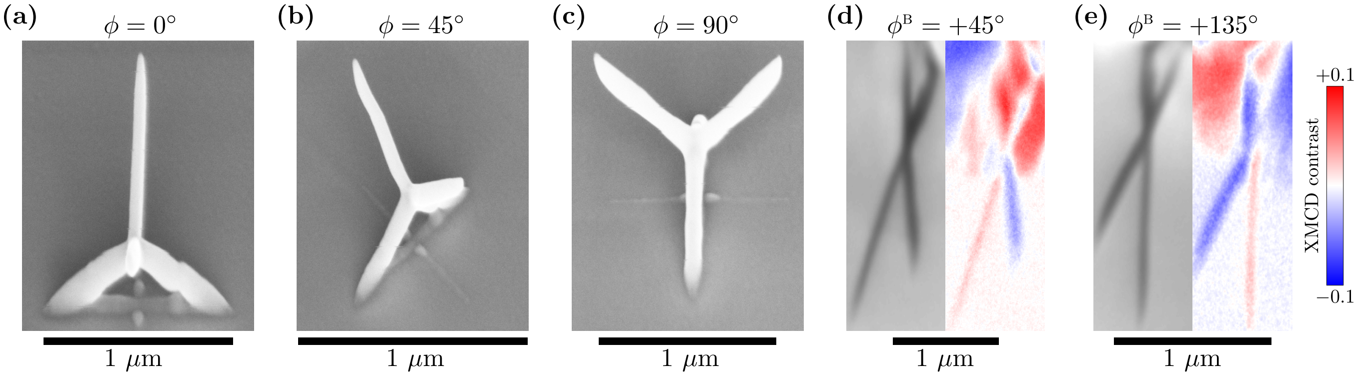

Supplementary Figure 6. **Magnetic tetrapod with top opening angle of 95° and bottom opening angle of 108°.** Scanning electron microscopy images of the tetrapod taken at different azimuthal angles: **(a)** $\phi = 0°$, **(b)** $\phi = 45°$ and **(c)** $\phi = 90°$. The total height of the tetrapod is 1.38 μm. It is constructed from linear segments with elliptical cross-section of 40 nm semi-minor and 72 nm semi-major axes. **(d-e)** PEEM image and the corresponding XMCD-PEEM shadow contrast image of the tetrapod. The images are taken at different azimuthal angles with respect to the incident X-ray beam: **(d)** $\phi = +45°$ and **(e)** $\phi = +135°$. The red-white-blue colour scheme represents the parallel, perpendicular and antiparallel alignment of the magnetization vectors with respect to the X-ray beam, respectively.

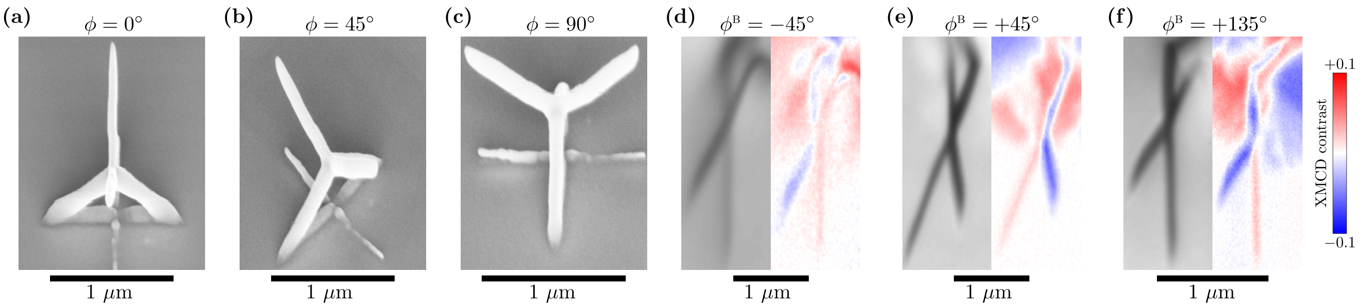

Supplementary Figure 7. **Magnetic tetrapod with top opening angle of 113° and bottom opening angle of 108°.** Scanning electron microscopy images of the tetrapod taken at different azimuthal angles: **(a)** $\phi = 0°$, **(b)** $\phi = 45°$ and **(c)** $\phi = 90°$. The total height of the tetrapod is 1.5 μm. It is constructed from linear segments with elliptical cross-section of 55 nm semi-minor and 67 nm semi-major axes. **(d-f)** PEEM image and the corresponding XMCD-PEEM shadow contrast image of the tetrapod. The images are taken at different azimuthal angles with respect to the incident X-ray beam: **(d)** $\phi = -45°$, **(e)** $\phi = +45°$ and **(f)** $\phi = +135°$. The red-white-blue colour scheme represents the parallel, perpendicular and antiparallel alignment of the magnetization vectors with respect to the X-ray beam, respectively.

Supplementary Section 4. Complex wireframe structures

We fabricated Co_3Fe semi-pyramid, diamond and cube wireframe structures by means of FEBID. The fabrication approach is the same as described for tetrapods. These complex wireframe structures are fabricated on top of a non-magnetic PtC pillar of 1.5 μm height to enable full magnetic characterization using XMCD-PEEM. These geometries are constructed of linear segments with the cross-section diameter of about 120 nm and 170 nm. The semi-pyramid structure is homeomorphic to 2-torus, see Supplementary Fig. 8. The diamond structure is homeomorphic to 3-torus, see Supplementary Fig. 9. The cube geometry is equivalent to 5-torus, see Supplementary Fig. 10.

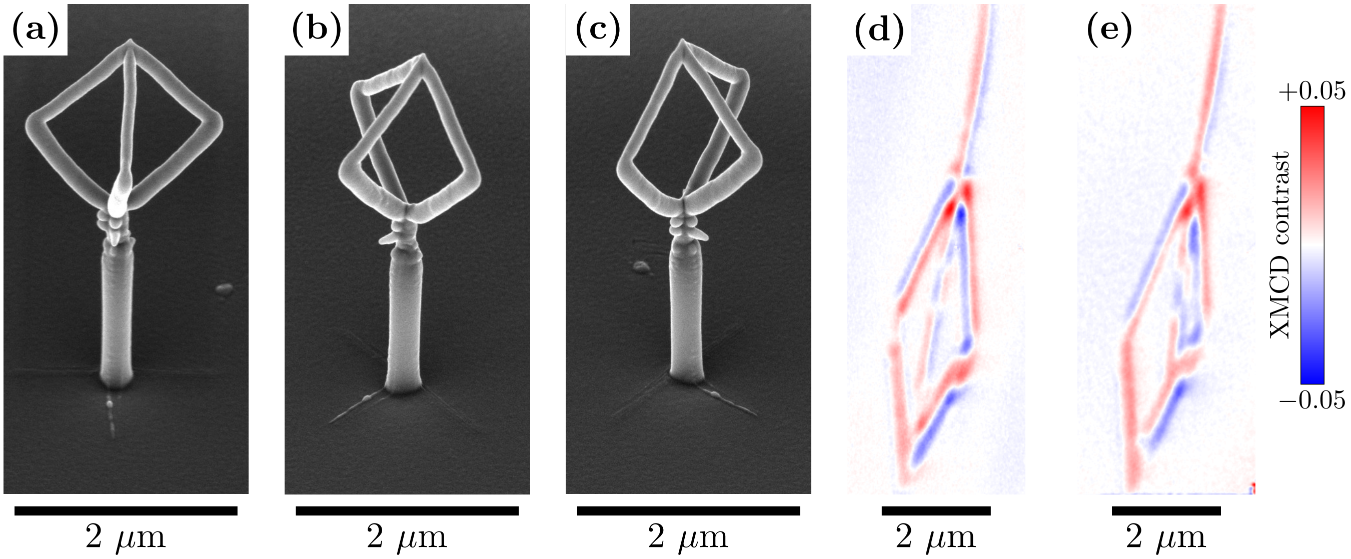

Supplementary Figure 8. **Magnetic semi-pyramid wireframe.** Scanning electron microscopy images of the semi-pyramid taken at different azimuthal angles: **(a)** $\phi = 0°$, **(b)** $\phi = -45°$ and **(c)** $\phi = 45°$. The total height of the semi-pyramid is 1.6 μm and it is grown on a non-magnetic PtC pillar with a height of 1.5 μm. The pyramid is constructed of linear segments with a cross-section diameter of about 130 nm and 180 nm. **(d-e)** XMCD-PEEM shadow contrast images of the semi-pyramid. The images are taken at different azimuthal angles with respect to the incident X-ray beam: **(d)** $\phi = 0°$ and **(e)** $\phi = -25°$. The red-white-blue colour scheme represents the parallel, perpendicular and antiparallel alignment of the magnetization vectors with respect to the X-ray beam, respectively.

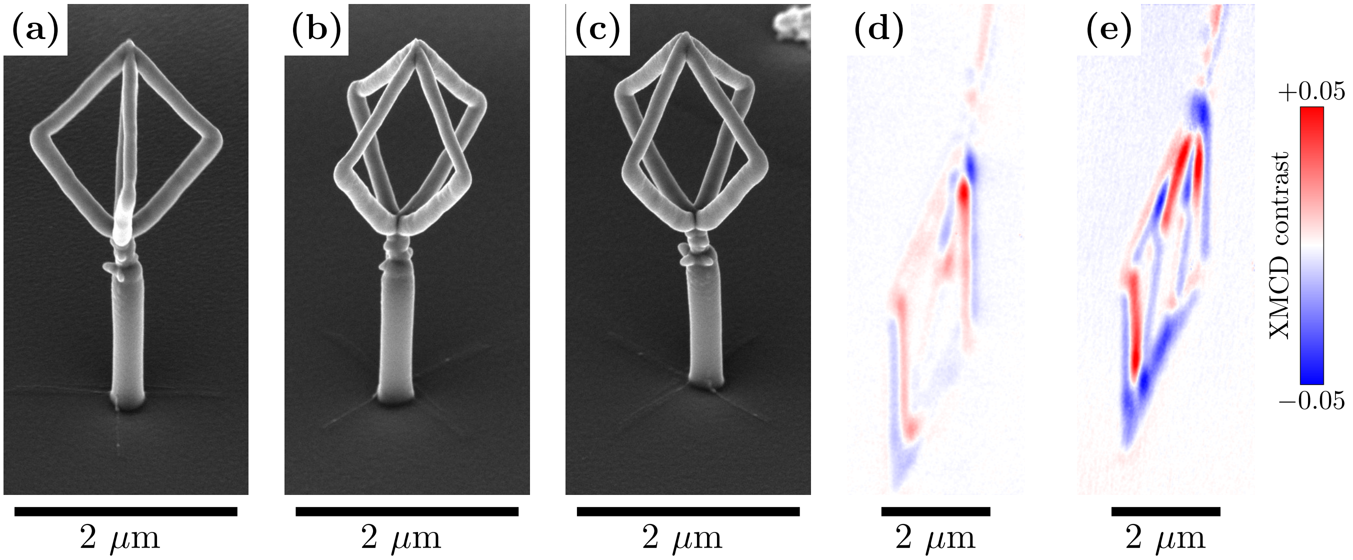

Supplementary Figure 9. **Magnetic diamond wireframe.** Scanning electron microscopy images of the diamond taken at different azimuthal angles: **(a)** $\phi = 0°$, **(b)** $\phi = -45°$ and **(c)** $\phi = 45°$. The total height of the diamond is 1.8 μm and it is grown on a non-magnetic PtC pillar with a height of 1.5 μm. The diamond is constructed of linear segments with a cross-section diameter of about 120 nm and 170 nm. **(d-e)** XMCD-PEEM shadow contrast images of the diamond. The images are taken at different azimuthal angles with respect to the incident X-ray beam: **(d)** $\phi = 0°$ and **(e)** $\phi = -25°$. The red-white-blue colour scheme represents the parallel, perpendicular and antiparallel alignment of the magnetization vectors with respect to the X-ray beam, respectively.

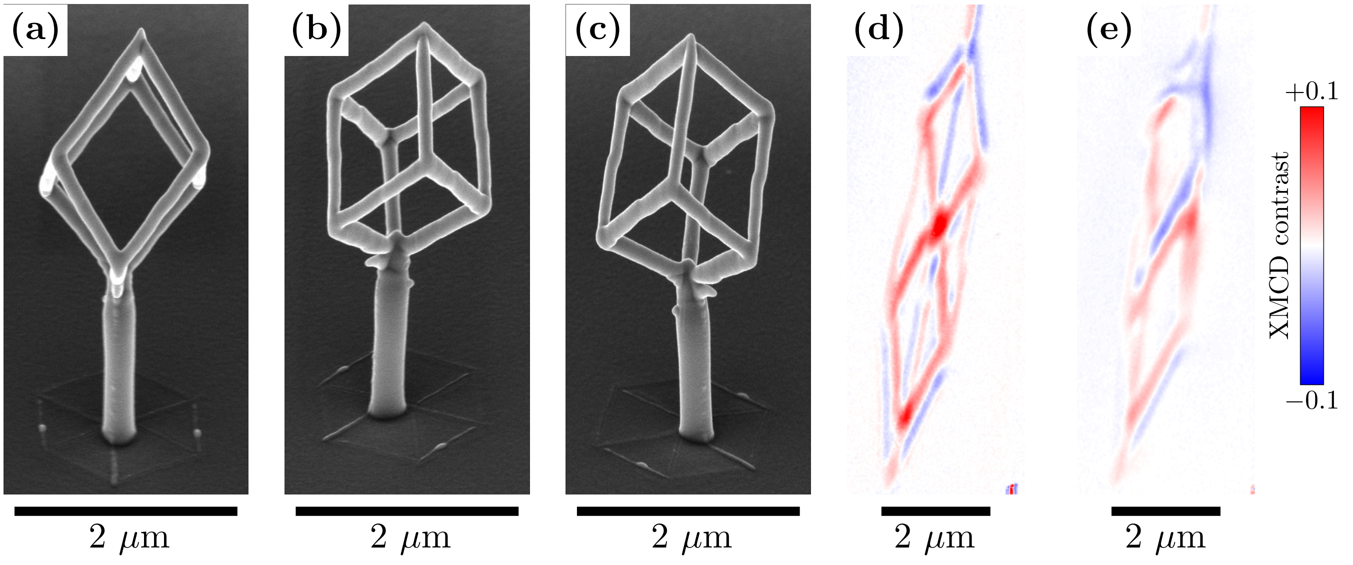

Supplementary Figure 10. **Magnetic cube wireframe.** Scanning electron microscopy images of the cube taken at different azimuthal angles: **(a)** $\phi = 0°$, **(b)** $\phi = -45°$ and **(c)** $\phi = 45°$. The total height of the cube is 2.3 μm and it is grown on a non-magnetic PtC pillar with a height of 1.5 μm. The cube is constructed of linear segments with a cross-section diameter of about 120 nm and 190 nm. **(d-e)** XMCD-PEEM shadow contrast images of the cube. The images are taken at different azimuthal angles with respect to the incident X-ray beam: **(d)** $\phi = 0°$ and **(e)** $\phi = -25°$. The red-white-blue colour scheme represents the parallel, perpendicular and antiparallel alignment of the magnetization vectors with respect to the X-ray beam, respectively.

Supplementary Section 5. XMCD-PEEM characterization

After fabrication, all tetrapods were imaged by means of X-ray magnetic circular dichroism photoelectron emission microscopy (XMCD-PEEM) at BESSY II (beamline UE49-PGM, Helmholtz-Zentrum Berlin, Germany). Before imaging, we investigated X-ray absorption spectra (XAS) at room temperature at Co and Fe absorption edges. The spectra were taken of the FEBID-grown tetrapod structures and co-deposited area around tetrapods, see Supplementary Fig. 11a and b. XAS data reveal that imaging at either Co or Fe absorption edges will provide XMCD-PEEM images of strong contrast (Supplementary Fig. 11c-d and e-f). In our studies, we chose to image at Co L_3 edge (777 eV).

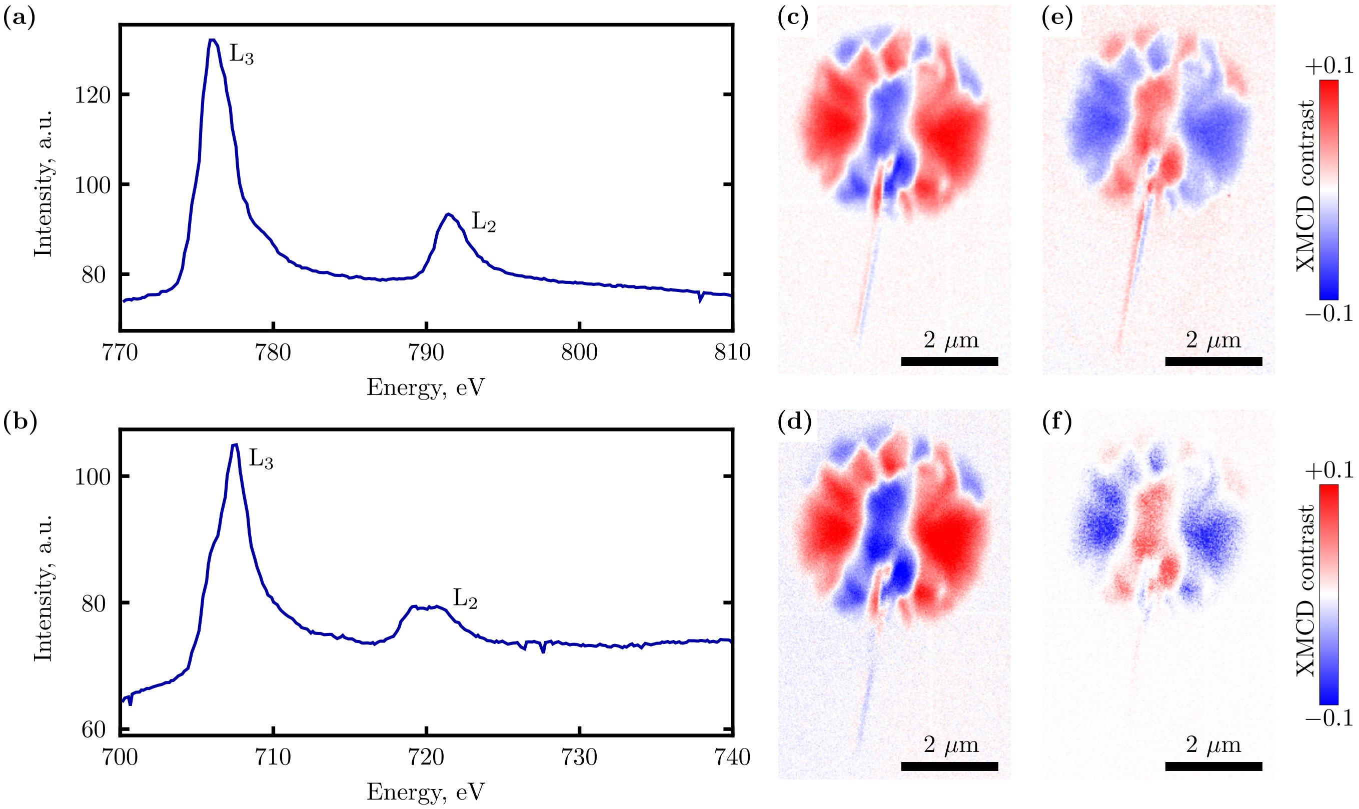

Supplementary Figure 11. **X-ray absorption spectra and XMCD-PEEM imaging at Co and Fe absorption edges.** X-ray absorption spectra taken at L_2 and L_3 absorption edges of **(a)** Co and **(b)** Fe. XMCD-PEEM images taken at **(c)** L_3 and **(e)** L_2 absorption edges of Co. XMCD-PEEM images taken at **(d)** L_3 and **(f)** L_2 absorption edges of Fe.

We chose the coordinate system in a way that the bottom part of each tetrapod is oriented along the $\hat{\boldsymbol{x}}$ axis. The azimuth angle ϕ is calculated with respect to this axis. For each tetrapod geometry, the XMCD-PEEM imaging is performed at multiple azimuth angles of $\phi = -45°$; $+45°$; $+135°$. Considering the high symmetry of the tetrapod structures, this allows reconstruction of the magnetization in the top and bottom segments.

The obtained XMCD-PEEM shadows for the tetrapod structures reveal the presence of a particular contrast, with one part of the tetrapod shadow being blue, while the other part is red. The red and blue contrasts in a shadow image represent magnetization vectors being antiparallel and parallel with respect to the direction of X-ray beam, respectively. The observed contrasts indicate a mostly homogeneous magnetization distribution in the respective segments. The white contrast is observed at the tetrapod center (at the join location) between the red and blue regions of the XMCD-PEEM shadow. Similar to the discussion in the main text, this white contrast is attributed to the antivortex-like distribution. The formation of surface antivortices is expected due to the 90° geometric rotation of the top and bottom parts of the studied tetrapods.

To interpret the measured XMCD-PEEM contrast, we evaluated the X-ray transmission intensities from the micromagnetically calculated magnetization distribution and compared them with XMCD-PEEM data. Namely, the intensity of the X-ray beam transmitted through the 3D structure depends on the magnetization distribution aligned

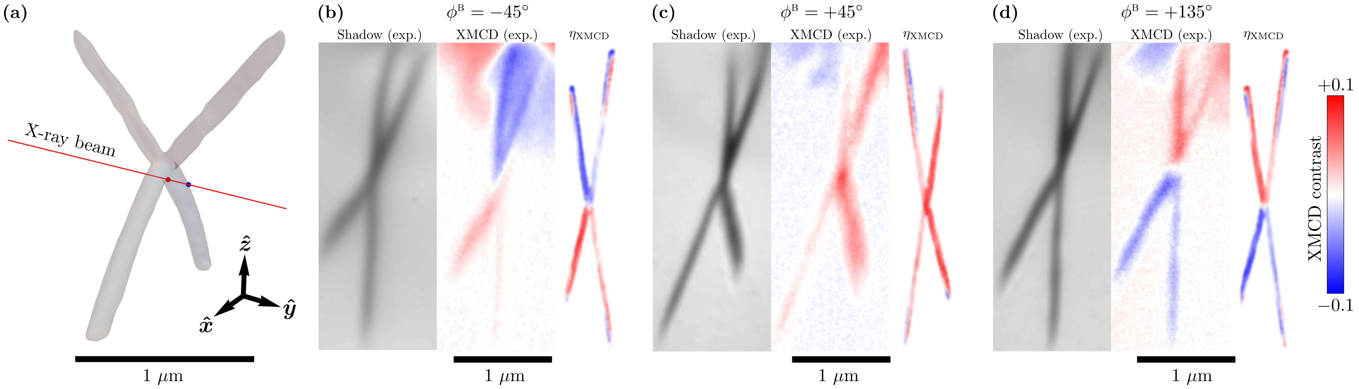

Supplementary Figure 12. **Calculation of the XMCD-PEEM shadow contrast for the experimental tetrapod geometry.** **(a)** Schematic representation of the X-ray beam intensity decrease as it passes through the tetrapod geometry. The red and blue spheres indicate an entry and exit points of the X-ray beam (red line) intersection with the tetrapod geometry. **(b–d)** show the experimental XMCD-PEEM shadow contrasts and their corresponding numerically calculated counterparts for different azimuth orientations of the incident X-ray beam: **(b)** $\phi = -45°$, **(c)** $\phi = +45°$ and **(d)** $\phi = +135°$, respectively.

along the beam for a particular beam circular polarization [**?**]

$$I^{\pm} = \exp\left[-\frac{k_0}{\lambda_{\text{Co}}\cos\theta_{\text{b}}}\int \mathrm{d}\boldsymbol{r}\,(1 \pm \delta_{\text{Co}}\boldsymbol{m}(\boldsymbol{r})\cdot\boldsymbol{k})\right], \tag{S7}$$

where k_0 is the coefficient related to the content of Co in the Co_3Fe tetrapod structure, $\lambda_{\text{Co}} = 82.8$ nm is the absorption length of Co at the L_3 edge, $\theta_{\text{b}} = 16°$ is the incident angle at which X-ray beam illuminates the structure (Supplementary Fig. 12a), δ_{Co} is the magnetic dichroism of circularly polarized X-rays with wave vector $\boldsymbol{k}$ and $\boldsymbol{m}(\boldsymbol{r})$ is the magnetization distribution inside the magnetic body along the direction of X-ray beam. Based on this Eq. 7 we calculate the resulting XMCD-PEEM shadow contrast through the relative intensity difference of photoemission electrons emitted from the tetrapod shadow for left I^- and right I^+ circular polarized X-rays

$$\eta_{\text{XMCD}} = \frac{I^+ - I^-}{I^+ + I^-}. \tag{S8}$$

The calculated XMCD-PEEM shadow contrast is encoded in a blue-white-red colors code associated with parallel, perpendicular and antiparallel beam alignments with respect to the magnetization, respectively [**?**]. Using Eq. 8 and the magnetization distribution obtained from the full-scale micromagnetic simulations, we calculate qualitatively the same shadows as were obtained experimentally for $\phi = -45°$, $\phi = +45°$ and $\phi = +135°$ azimuthal angles of the incident X-ray beam, see Supplementary Fig. 12b–d, respectively.

Supplementary Section 6. Magnetic hysteresis of a tetrapod structure

As discussed in the main text, the interconnection between the total vorticity of the surface magnetization distribution and the Euler characteristic of the geometry remains valid even when the object is exposed to an external magnetic field. To investigate the in-field evolution of the total surface magnetic vorticity, we perform micromagnetic simulations of the magnetic hysteresis using the GPU accelerated TetraMag code [? ?]. The calculations are performed for the experimental tetrapod geometry. The magnetic field is aligned along $\hat{\boldsymbol{z}}$ axis, $B = [-300; +300]$ mT. For each field value starting from $B = +300$ mT and field step of $\Delta B = -2$ mT, the equilibrium magnetic state is obtained by means of the conjugate gradient energy minimization approach. The resulting hysteresis of the m_z magnetization component with respect to the external magnetic field is shown in Supplementary Fig. 13a . The equilibrium magnetic states with their corresponding azimuthal and polar angle distributions are shown in Supplementary Fig. 13b–e for $B = +300$; 0; -20; -26 mT, respectively. The analysis of the magnetic states indicates the presence of four vortices and two antivortices. Thus, the total vorticity calculated over the tetrapod surface remains $Q^{\Sigma} = +2$. Moreover, the total vorticity remains equal to the Euler characteristic even when the tetrapod is exposed to the field of 10 T. In this case, we observe two vortices in the connection region of the tetrapod (Fig. 13f). The magnetic state in the cross-section of the central part of the experimental tetrapod geometry with the indication of the surface antivortices and homogeneously magnetized interior in absence of magnetic fields is shown in Fig. 14.

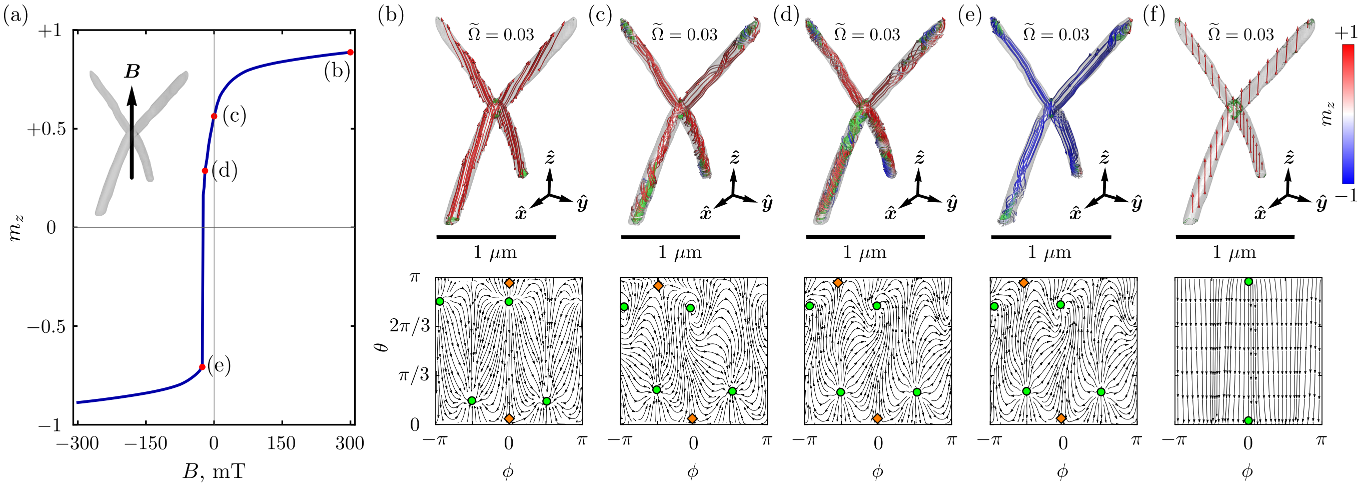

Supplementary Figure 13. **Magnetic hysteresis of the tetrapod.** **(a)** Hysteresis curve calculated for the experimental tetrapod geometry. The tetrapod is exposed to the magnetic field aligned along $\hat{\boldsymbol{z}}$ axis. The inset depicts the direction of the field with respect to the tetrapod alignment. Equilibrium magnetic states calculated for the tetrapod exposed to the magnetic field of **(b)** $B = +300$ mT, **(c)** $B = 0$ mT, **(d)** $B = -20$ mT and **(e)** $B = -26$ mT. The streamlines indicate the internal magnetization distribution inside the tetrapod volume. The red-white-blue color scheme represents the m_z magnetization components. Green regions show isosurfaces of the topological charge density. The bottom panels show the surface magnetization distribution in azimuthal and polar coordinates of the corresponding angular projection of the magnetization on the unit sphere. Green disks indicate the positions of vortices, orange diamonds denote the position of antivortices. **(f)** Equilibrium magnetic state of the tetrapod exposed to the magnetic field $B = +10$ T. The object accommodates two vortices in the central region of the tetrapod.

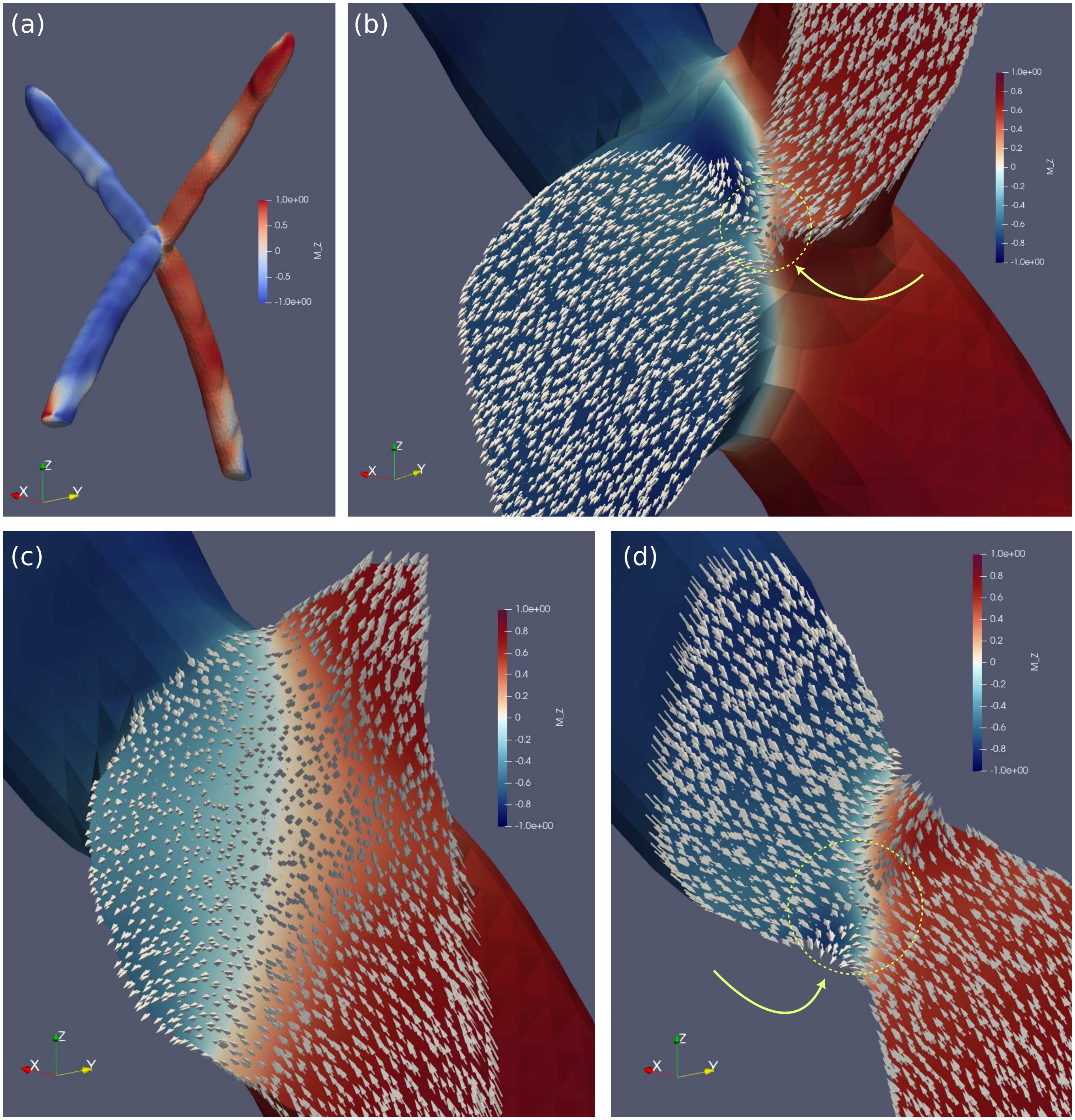

Supplementary Figure 14. **Interior of the tetrapod near surface antivortices.** **(a)** 3D view of the tetrapod (experimental geometry). Here, and in other panels, the colour code defines the m_z component of magnetization. **(b)** Cross-section of the tetrapod from the same view point as in panel (a) in the vicinity of the surface antivortex below the point at the surface corresponding to the antivortex core. White arrows correspond to the magnetization direction at the cross-section. The location of the antivortex in the cross-section is shown by a bright-green arrow and dotted circle. In this cross-section, the antivortex core as the point at the Bloch line can be clearly identified. **(c)** Cross-section of the tetrapod in the central part. The center of the cross-section is homogeneously magnetized along the cross-section normal, and the antivortex core cannot be identified. **(d)** Same as **(b)** in the vicinity of the second surface, where the antivortex core can be clearly identified in the cross-section.

Supplementary Section 7. Magnetic wireframes: examples

In addition to the tetrapod geometry analyzed in the main text, we investigate numerically other magnetic wireframes, which will be discussed in this section. The following simulations are performed for the material parameters of Co_3Fe, introduced in the main text.

A. Six-arm star geometry

A planar wireframe structure with six-fold symmetry consisting of six arms is considered. For each particular geometry, the radius and length of the arms is chosen in the range from 10 to 50 nm and from 50 to 800 nm, respectively. Having the surface topologically equivalent to a sphere, this geometry forces the appearance of six vortices at the ends of each arm and four antivortices. We relaxed the magnetic state starting from a uniform alternating direction in each of 60° segments containing one arm. In equilibrium, there are four antivortices in the connecting region in the center, which are connected by two Bloch lines in a pairwise manner (bulk antivortex texture). An exemplary simulation result is shown in Fig. 15. Geometries of other sizes give the qualitatively same picture.

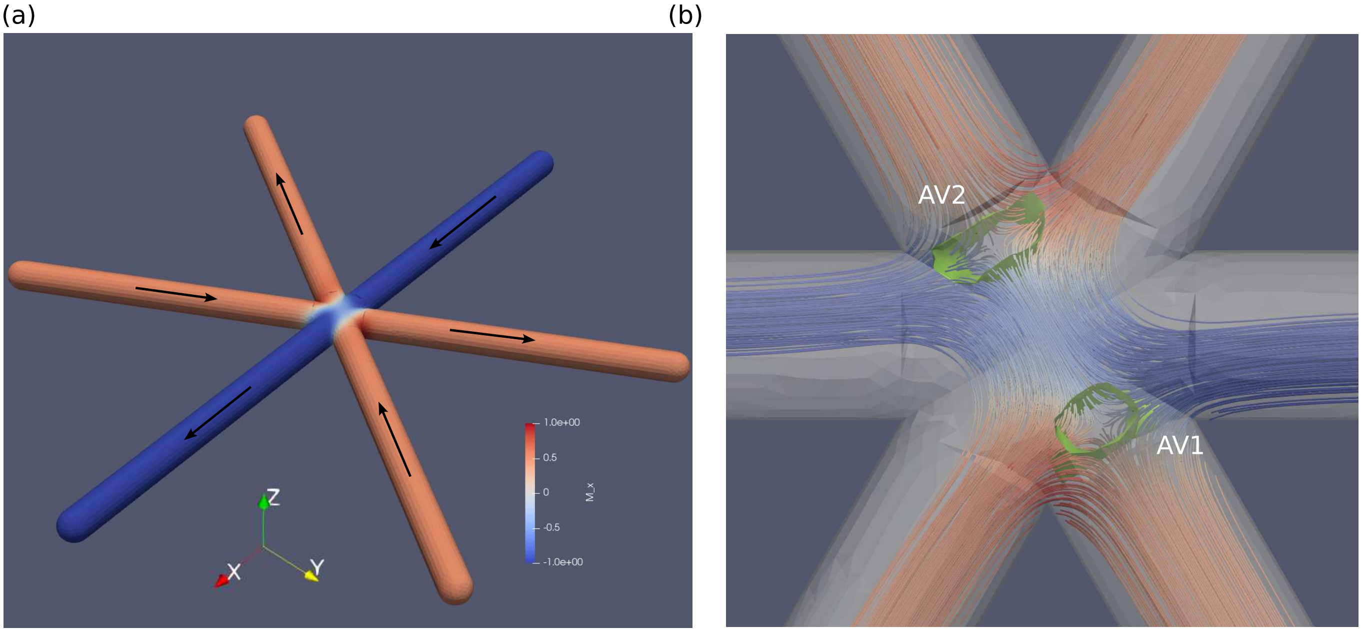

Supplementary Figure 15. **Magnetic texture in a six-arm star.** **(a)** The geometry of a six-arm star with the arm length of 200 nm and arm radius of 10 nm. Blue-red color corresponds to the $\hat{\boldsymbol{x}}$ component of magnetization. Each arm ends with a vortex texture. Black arrows indicate magnetization direction for each of the arms. **(b)** Streamlines of magnetization around each antivortex (view along $-\hat{\boldsymbol{z}}$ axis). Color code is the same as in panel (a). Topological charge flux density distribution around antivortices, indicated by green isosurfaces ($\widetilde{\Omega} = 0.63$).

B. Tripod

A tripod consists of three nanowire segments connected in one junction area. Such geometry was intensively studied experimentally as an artificial building block for 3D spin ice [**?**]. In simulations, each nanowire segment has a radius of 58 nm and a length of 1.3 μm. An azimuth angle between segments is 120° with the opening angle of 80°. Micromagnetic simulations were started from different initial states, including random state, as well as homogeneous magnetic distributions, aligned along $\hat{\boldsymbol{x}}$ and $\hat{\boldsymbol{z}}$ axes. After relaxation, the equilibrium magnetization distribution obtains three surface vortices with $Q_{\mathrm{v}} = +1$ at the ends of line segments, that slowly transform into the homogeneous state near the connection area, see Supplementary Fig. 16a and (b). The geometric frustration of the tripod results in two wires magnetized towards the junction and one out of the junction. This leads to the formation of the surface

antivortex, see Supplementary Fig. 16c. Thus, the total vorticity calculated for the tripod surface is

$$Q^{\Sigma} = 3Q_{\mathrm{v}} + 1Q_{\mathrm{av}} = 2, \tag{S9}$$

which is in line with the Euler characteristic of a sphere. This magnetization distribution was reported recently for a GdCo tripod imaged by means of soft X-ray laminography [?]. We note that at the opposite side from the antivortex, there is a volume region of high topological charge flux density Ω, which is related to the curling of the magnetization streamlines, see Supplementary Fig. 16c. However, this is not a topological defect because of the possibility to map the resulting texture onto a uniform vector field.

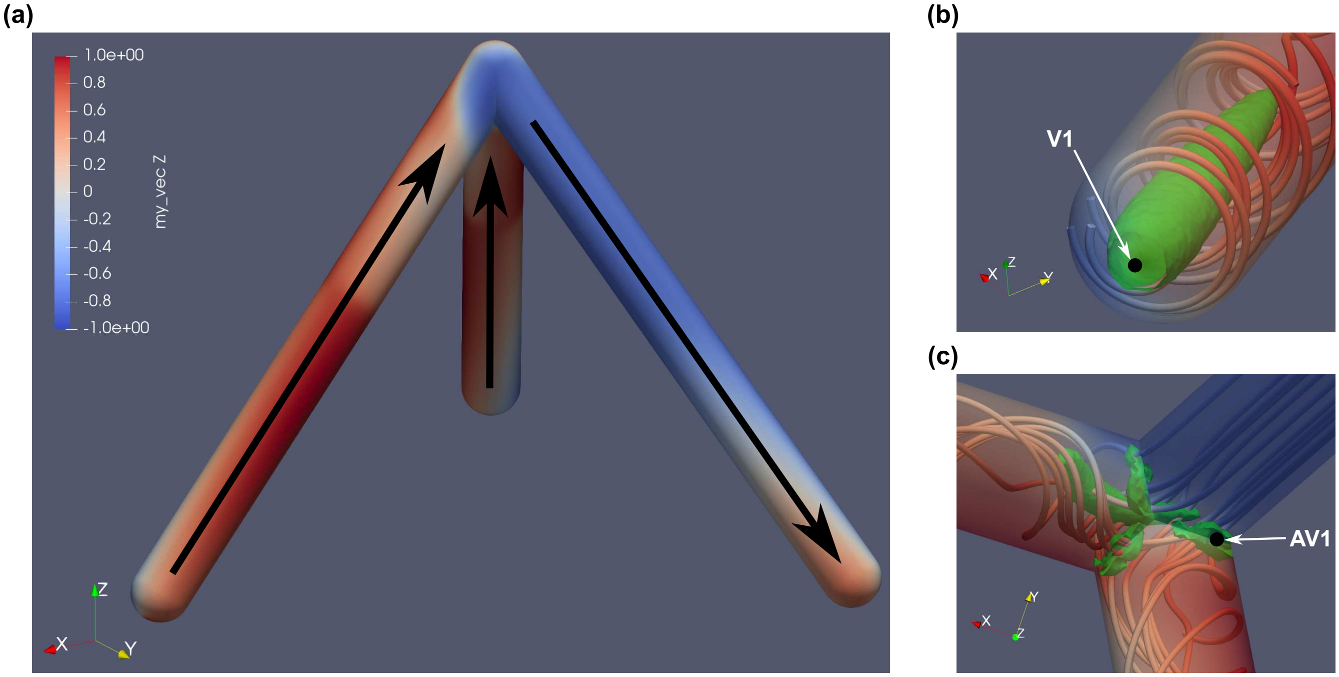

Supplementary Figure 16. **Magnetic texture in a tripod.** **(a)** Magnetization distribution in the tripod geometry. The color contrast corresponds to the $\hat{\boldsymbol{z}}$ component of magnetization being red when pointing up and blue when pointing down. The direction of magnetization in each of the wires is marked by black arrows. **(b)** Surface vortex state at the end of a wire. Magnetization streamlines are shown as well. The green isosurface indicates the distribution of the topological charge flux density with $\widetilde{\Omega} = 0.053$. The position of the vortex on the tripod surface is marked by a black sphere and labeled as "V1". **(c)** Surface antivortex in the junction region. Streamlines show the magnetization distribution at the junction. The position of the antivortex is marked by a black sphere and labeled as "AV1".

C. Pentapod

Here, we study the magnetic state of the wireframe architecture with five arms, shown in Supplementary Fig. 17a. The radius of each of the arms, as well as the ending, is 58 nm, arm length is 1300 nm. The opening angle of the bottom arms is 100°. Supplementary Fig. 17a shows the equilibrium state of the pentapod with three wires magnetized downward (mainly colored in blue), and two arms magnetized upward (mainly colored in red). In equilibrium, magnetization at the ends of the wires forms five vortices. The surface topology forces formation of three antivortices, which can be found in the junction region. Thus, the total vorticity calculated over the pentapod surface is

$$Q^{\Sigma} = 5Q_{\mathrm{v}} + 3Q_{\mathrm{av}} = 2 \equiv \chi_{\mathrm{sphere}}, \tag{S10}$$

where χ_{sphere} is the Euler characteristic of a sphere. This is in agreement with Poincaré–Hopf theorem [?].

Supplementary Figs. 17b–d show the magnetization streamlines around each antivortex. The topmost antivortex labeled as "AV1" (polarity directed *into* the volume) has a direct connection with one upper arm and three nearest bottom arms via these streamlines. It is separated from the fifth leg by the almost homogeneously magnetized volume. Antivortex "AV2" (polarity directed *outside* the volume) is located at the bottom part of the pentapod junction. This

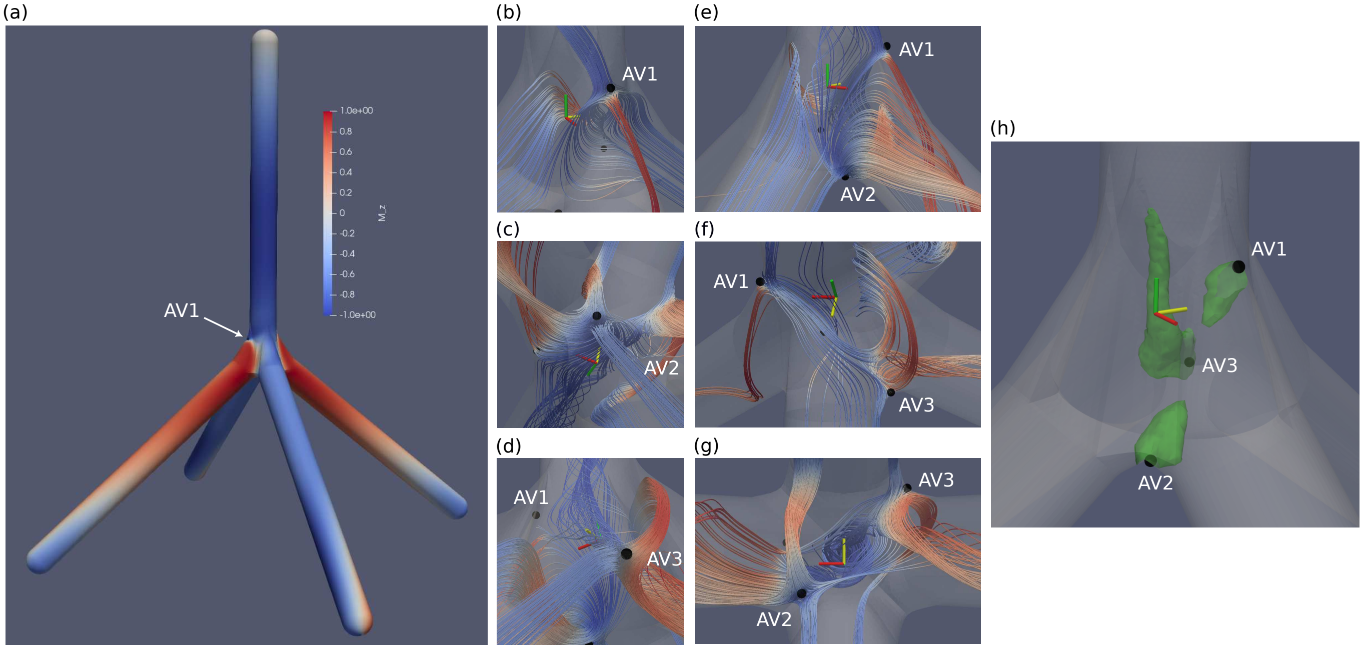

Supplementary Figure 17. **Magnetic texture in a pentapod.** (a) The shape and the calculated magnetic state of the pentapod. Blue-red color corresponds to the $\hat{\boldsymbol{z}}$ component of magnetization. Each wire ends with a vortex texture. The position of one out of three antivortices is marked by a small black sphere and labeled as "AV1". (b–d) Streamlines of magnetization around each of three antivortices marked by black spheres and labeled as "AV1", "AV2" and "AV3". Color code is the same as in panel (a). Red, yellow and green rods in the origin show directions of $\hat{\boldsymbol{x}}$, $\hat{\boldsymbol{y}}$, $\hat{\boldsymbol{z}}$ axes, respectively. (e–g) Streamlines of magnetization connecting each pair of antivortices. Color code is the same as in panel (a). (h) Topological charge flux density distribution around antivortices (isosurfaces of $\widetilde{\Omega} = 0.056$ are shown with green color).

antivortex is directly connected by streamlines with four bottom arms. The third antivortex "AV3" (polarity directed *outside* the volume) is located slightly higher than "AV2" at the opposite side of the pentapod. Similarly to "AV1", it is connected by streamlines with three bottom arms and the top arm of the pentapod.

We note that behind each of the surface antivortices, there is a sample volume, which is almost homogeneously magnetized in the direction, parallel to the surface at which the antivortex is located. There are no Bloch lines crossing the complete volume of the pentapod (see also elongated regions where the maxima of the topological charge flux density are located around antivortices; Supplementary Fig. 17h). Supplementary Figs. 17e–g show pairwise connections between antovortices via stream lines. We note that the weakest connection is observed between "AV2" and "AV3" [Supplementary Fig. 17g], where the connecting streamlines bend around one of the pentapod arms.

D. Pyramid

The five-vertex pyramid is constructed out of eight wires with radius of 58 nm and length of 1300 nm (Supplementary Fig. 18a). The object possesses five holes and is topologically equivalent to a 4-torus (torus with four holes). For this object, the Euler characteristic $\chi_{4\text{-torus}} = -6$.

A torus with one hole is characterized by $\chi = 0$ and supports the locally uniform magnetization along the tangential direction, which is topologically trivial [**?** **?**]. Tori with higher number of holes can be arranged differently in $\mathbb{R}^3$, which determines the stable magnetization configurations and types of magnetic solitons observable at their surfaces.

Starting from a random initial distribution of magnetization and performing energy minimization for the pyramid, we obtain the state with two antivortices at the top pyramid vertex. Both antivortices are placed asymmetrically at the outer and inner surfaces of the connecting region, see Supplementary Fig. 18b. They are connected by a curled Bloch line. Magnetization in the pyramid's base forms a closed counter-clockwise flux with strong distortions near the connection of pyramid edges. At the edges, we identify four antivortices with $Q_{\text{av}} = -1$. At the opposite side from the antivortex, there is a region of high topological charge flux density Ω, related to the curling of the magnetization streamlines similar to a quarter of an antivortex, see Fig. 18c. However, it is not a topological defect because of the possibility to map it onto a uniform vector field. We note that all four curlings observed near the bottom vertices cannot be combined to a complete antivortex because of their random placement at the left or right sides from the

respective upper arms. Thus, the total vorticity calculated on the pyramid's surface is

$$Q^{\Sigma} = 6Q_{\text{av}} = -6 \equiv \chi_{\text{4-torus}}. \tag{S11}$$

We note that the pyramid is a geometrically frustrated object and possesses multiple states with close or same magnetic energy. For example, a magnetic texture without antivortices at the top vertex has higher energy than the discussed above.

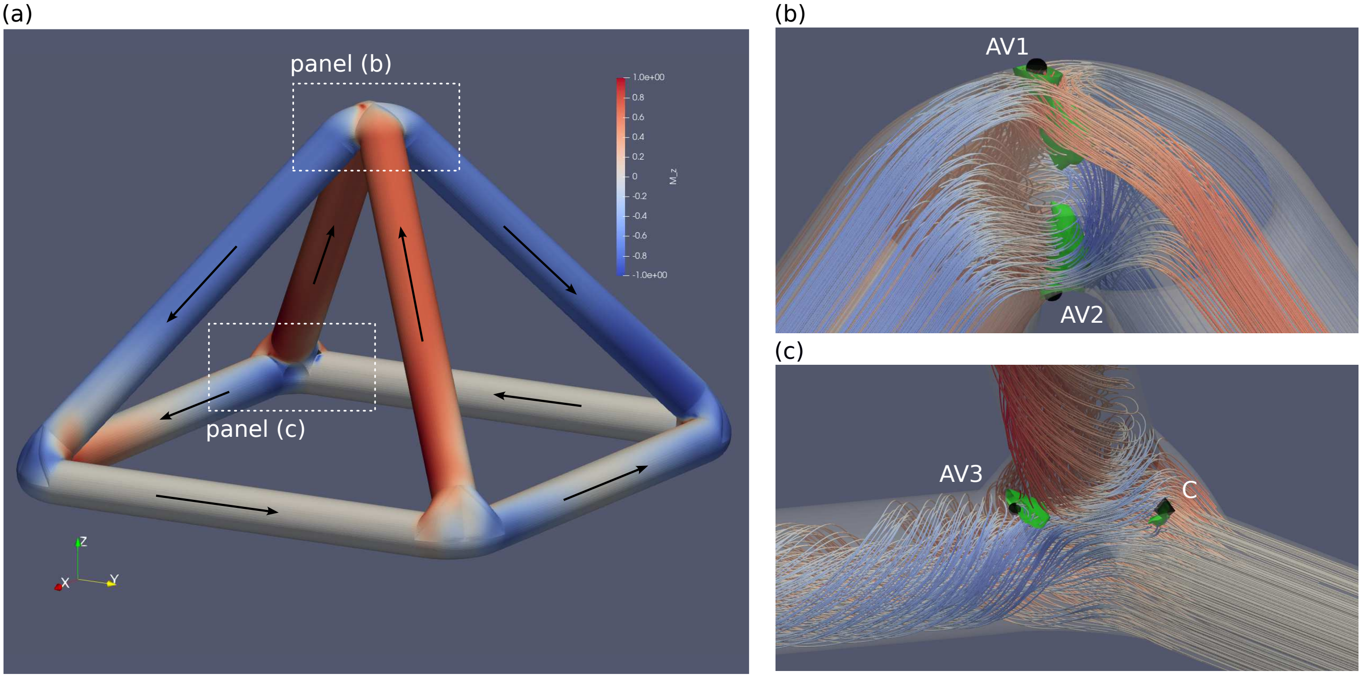

Supplementary Figure 18. **Magnetic texture in a wireframe pyramid.** **(a)** Magnetic state of the wireframe pyramid. The blue-red color code corresponds to the $\hat{\boldsymbol{z}}$ component of magnetization. The direction of magnetization in each arm is marked by black arrows. Regions depicted in panels **(b)** and **(c)** are highlighted by white dashed rectangles. **(b)** Magnetization streamlines at the top vertex of the pyramid (along $-\hat{\boldsymbol{x}}$ direction). The color code of the streamlines is the same as in panel (a). Black dots labeled as "AV1" and "AV2" mark positions of two antivortices. Green surfaces indicate the topological charge flux density distribution around these two antivortices ($\widetilde{\Omega} = 0.92$). **(c)** The antivortex labeled "AV3" (black sphere) and curling of magnetization at the side labeled as "C" near the bottom pyramid vertex. Magnetization direction is shown by streamlines. The color code of the streamlines is the same as in panel (a). Green isosurfaces indicate the region of the topological charge flux density with $\widetilde{\Omega} = 0.61$.

E. Cube

The wireframe cube is constructed of twelve wires, see Supplementary Fig. 19a. Each wire has radius of 58 nm and lenth of 1.3 μm. Such wireframe geometry has six holes and is topologically equivalent to a 5-torus (torus with five holes). This object has the Euler characteristic $\chi_{\text{5-torus}} = 2 - 2g = -8$ [?], where $g = 5$ is the genus of the surface (number of holes). In equilibrium, we obtain the magnetic state with 12 mainly homogeneously magnetized line segments, that form six vortex loops in each of square faces of the cube, see Supplementary Fig. 19a. Such configuration is characterized by the geometric frustration being similar to the one of the tripod geometry discussed in Supplementary Section 7 B. Thus, surface antivortex states appear at the vertices of the cube resulting in the total vorticity

$$Q^{\Sigma} = 8Q_{\text{av}} = -8 \equiv \chi_{\text{5-torus}}. \tag{S12}$$

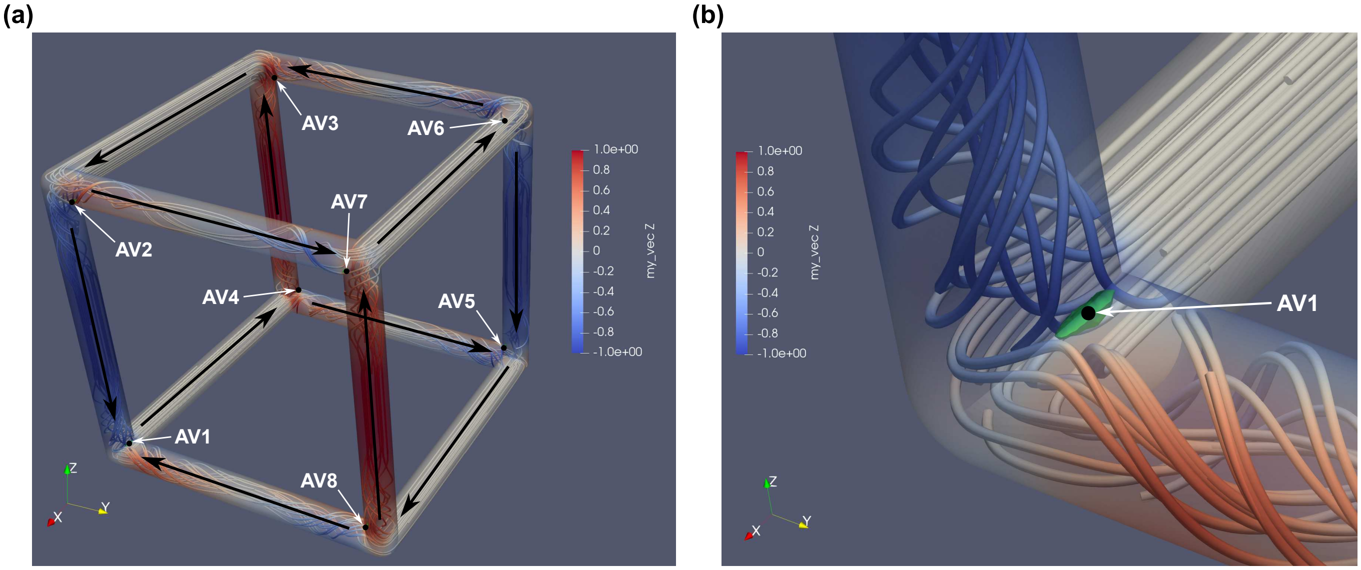

Supplementary Figure 19. **Magnetic texture in a wireframe cube.** **(a)** Equilibrium magnetization distribution in the cube visualized via streamlines. The color code corresponds to the $\hat{\boldsymbol{z}}$ component of magnetization being red when pointing up and blue when pointing down. The direction of magnetization in each of the segments is marked by black arrows. Positions of surface antivortices are marked by a black sphere. **(b)** The surface antivortex state in the vertex region between three line segments of the cube. The green isosurface indicates the distribution of the topological charge flux density with $\widetilde{\Omega} = 0.44$. The position of the antivortex is marked by a black sphere and labeled as "AV1".

Supplementary Section 8. Stray fields in wireframe structures

In this section, we provide details on the topology of stray fields created by magnetic wireframes. We performed reconstruction of stray fields emerging in the experimental tetrapod (Fig. 6 of the main text and the corresponding discussion). The interpretation of the experiment-related data shown in the main text is based on the analysis of stray fields of ideal tetrapods. As ideal geometries, we constructed cylinder-based tetrapods with different rotation between the planes accommodating upper and lower legs: 0°, 45° and 90° (Supplementary Fig. 20a,f and k). Each row in Supplementary Fig. 20 depicts selected perspectives of the three-dimensional magnetic stray fields emerging for each tetrapod geometry. The data is represented by isosurfaces of B_z providing insight in the spatial distribution of the magnetic stray fields.

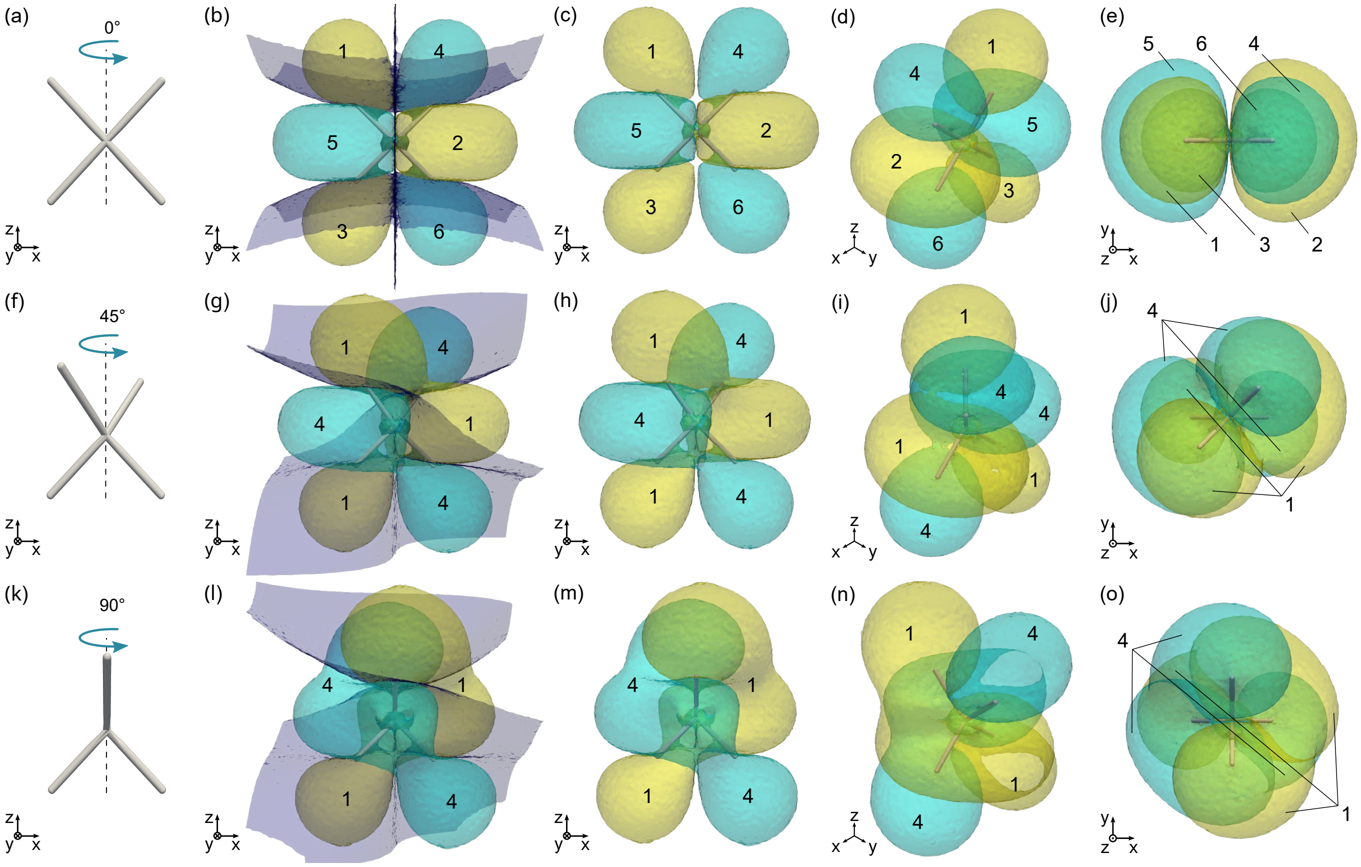

Supplementary Figure 20. **Magnetic stray fields in tetrapod geometries.** The image depicts magnetic stray fields of ideal tetrapod geometries (geometry is shown in panels **(a, f, k)**), which differ by the rotation angle between planes accommodating upper and lower legs of the tetrapod. **(b–e)** show isosurfaces of the B_z component for different viewpoints of the magnetic field calculated for the geometry shown in panel (a). **(g–j)** and **(l–o)** represent the corresponding viewpoints for the 45° and 90° tilted geometries. The isosurfaces with $B_z/B_{z,\max}$ values of 3.4×10^{-4}, -3.4×10^{-4}, and 0 are shown in yellow, cyan, and dark blue, respectively. The isosurface $B_z/B_{z,\max} = 0$ is shown only in panels (b), (g), and (l) for ease of interpretation of the data presented in other panels. In panels (b–e), (g–j), and (l–o) disconnected regions are indexed as 1–3 and 4–6 for $B_z/B_{z,\max} > 0$ and $B_z/B_{z,\max} < 0$, respectively. $B_{z,\max}$ is 591 mT.

A. Symmetry of stray field isosurfaces in homeomorphic tetrapods

The most symmetric tetrapod (model with the rotation angle 0°; Supplementary Fig. 20a) displays a $B_z = 0$ isosurface which is highly symmetric with respect to the centre of the tetrapod and $\hat{\boldsymbol{x}}$, $\hat{\boldsymbol{y}}$ and $\hat{\boldsymbol{z}}$ axes (Supplementary Fig. 20b). This isosurface indicates the regions where the field is genuinely horizontal and parallel to the $\hat{\boldsymbol{x}}\hat{\boldsymbol{y}}$-plane. There are three distinguishable surfaces where this occurs: the symmetry plane $\hat{\boldsymbol{y}}\hat{\boldsymbol{z}}$, and the upper and bottom hyperbolic-like surfaces.

The tetrapod models, which are characterized by the non-zero rotation angle (Supplementary Fig. 20f,k), are characterized by stray fields without mirror symmetry with respect to the $\hat{\boldsymbol{x}}$, $\hat{\boldsymbol{y}}$, i.e. with respect to $\hat{\boldsymbol{x}}$ and $\hat{\boldsymbol{y}}$ axes in $B_z = 0$. The isosurface $B_z = 0$ is widened and twisted around the $\hat{\boldsymbol{z}}$-axis in a way that modifies its morphology to a single connected surface (Supplementary Fig. 20g–l).

This finding suggests that modifications of the geometry of the tetrapod can have a profound impact on the topology of the stray field.

B. Classification of stray field topology

Stray field data obtained for the most symmetric tetrapod (rotation angle 0°; Supplementary Fig. 20a) reveals that $B_z > 0$ and $B_z < 0$ isosurfaces consist of six disconnected lobe-shaped regions (Supplementary Figs. 20c–d). As there are three disconnected regions in space where the field has positive vertical component B_z and three other regions where B_z is negative, the genus of each of these isosurfaces is 3. These isosurfaces can adopt complex shapes and can overlap depending on the view point. For clarity we present isosurfaces with a certain degree of transparency. At the overlapping regions, isosurfaces indicated with different colors (yellow and cyan) are seen in greenish tones (Supplementary Fig. 20e). We have introduced labels 1–3 and 4–6 in every panel to index the disconnected regions of $B_z > 0$ and $B_z < 0$ isosurfaces, respectively. Gradients of magnetic stray fields will appear in-between neighbouring regions with opposite sign, such as between lobes 1 and 5.

When the tetrapod geometry possesses a non-zero rotation angle (Supplementary Fig. 20f and k), magnetic stray fields preserve certain symmetry around $\hat{\boldsymbol{z}}$-axis (Supplementary Figs. 20h and m). The number of disconnected regions is reduced to only one for $B_z > 0$ and $B_z < 0$ isosurfaces by means of merging regions number 3 and 2 into 1 as well as 6 and 5 into 4 (compare Supplementary Fig. 20c and Supplementary Fig. 20h and m). This merge between regions is more evident in the case shown in Supplementary Fig. 20m and confirms that these isosurfaces have a topology with genus 1. The increase of the rotation angle in the tetrapod model lowers the genus of each stray field isosurface from 3 to 1. This is accompanied by the gradual merge of regions. Thus, while tetrapods shown in Supplementary Figs. 20a and k are homeomorphic, they obtain stray field isosurfaces of different topology.

C. Inhomogeneous stray fields: Field orientability for applications

Genus 3 indicates that there are three disconnected regions in space where the field has positive vertical component B_z and three other regions where B_z is negative. Hence, tetrapod structures can provide strong gradients in the magnetic near field, which is relevant for applications in small scale robotics [**? ?**], pinning/trapping of magnetic objects [**? ?**] and manipulating energy landscapes for superconducting electronics [**?**]. In particular, the possibility to change the geometry of the tetrapod (e.g., different rotation angle) allows tailoring the spatial profile of the magnetic stray field and designing its gradient. For instance, disconnected lobes in Supplementary Fig. 20e (genus 3) offer a highly anisotropic magnetic stray field orientated along the $\hat{\boldsymbol{x}}$-axis. In contrast, the lobes in Supplementary Fig. 20o (genus 1) indicate that this tetrapod geometry can be used to realize regions with a well-defined vertical component of the stray field that covers large angular range around the tetrapod.

D. Geometrically-induced robustness of the stray field of tetrapods exposed to an external magnetic field

Stability against perturbations is a desirable feature in applications. In Supplementary Section 6 we showed that vortices in tetrapods are stable even when the tetrapod is exposed to a strong magnetic field. Here we show robustness of stray fields in tetrapods exposed to an external magnetic field.

We performed calculation of stray fields for the experimental tetrapod geometry when it is exposed to a static magnetic field of 500 mT in the $\hat{\boldsymbol{x}}\hat{\boldsymbol{y}}$ plane. Supplementary Fig. 21f shows the resulting stray field profile visualized via isosurfaces of B_z (Supplementary Fig. 21g–j), following the same analysis as in Fig. 5 of the main text and Supplementary Fig. 20. On the other hand, Supplementary Fig. 21a shows the resulting stray field profile visualized via isosurfaces of B_z (Supplementary Fig. 21b–e) in a magnetic state with no field.

We identify the same symmetries of the magnetic stray field as for the case of the tetrapod structure at remanence (compare Supplementary Fig. 21 and Supplementary Fig. 20m). The presence of a single region for each $B_z > 0$ (yellow) and $B_z < 0$ (blue) isosurfaces confirms the identical topology of the magnetic stray field (genus 1) with non-zero external magnetic field. The $B_z = 0$ isosurfaces consists of a single surface twisting around the $\hat{\boldsymbol{z}}$-axis of the tetrapod.

Supplementary Section 9. Supplemetary Videos

Supplemetary Video 1. This video visualizes the ansatz formula for the transformation between bulk and surface antivortices, which is presented in Supplementary Section 1 of the Supplementary Information.

Supplemetary Video 2. This video shows the real-time energy minimization process for a tetrapod with a variable rotation angle. For information about the model and parameters, see Supplementary Section 2 E of the Supplementary Information.

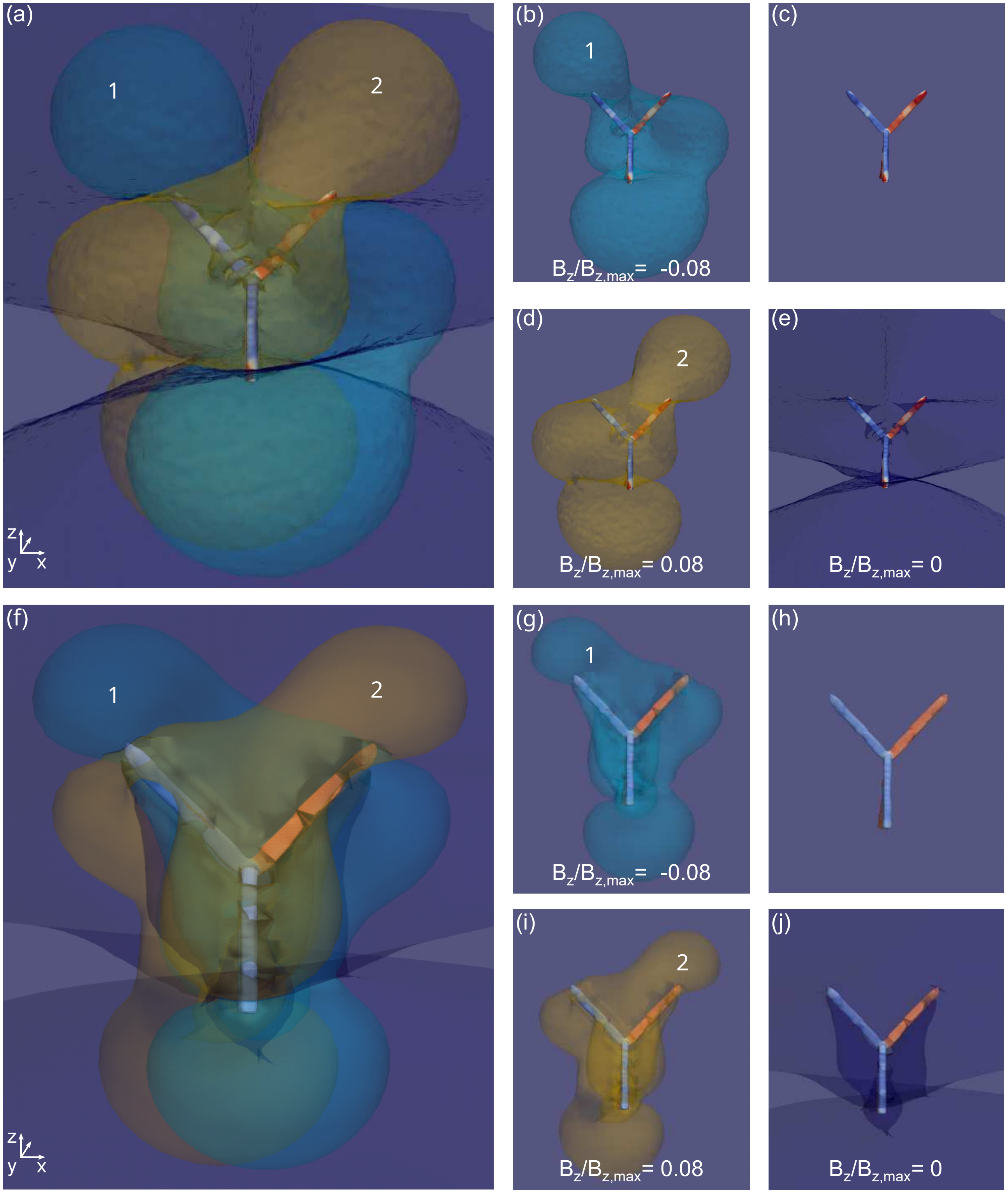

Supplementary Figure 21. **Stray fields of the experimental tetrapod geometry exposed to the external magnetic field of 0 mT (a–e) and 500 mT (f–j) applied in $\hat{x}\hat{y}$-plane.** The figure shows only the stray field of the magnetic texture, which is modified by the external magnetic field, but it is not superimposed with the external magnetic field. **(a)** Selected isosurfaces of B_z component of the stray field for an external applied magnetic field of 0 mT. **(b-e)** A decomposition of isosurfaces shown in panel (a) into isosurfaces with **(b)** $B_z > 0$ (yellow), **(d)** $B_z < 0$ (blue), **(c)** the magnetic state in the tetrapod and **(e)** isosurface with $B_z = 0$ (dark blue). **(f)** Selected isosurfaces of B_z component of the stray field for an external applied magnetic field of 0 mT. **(g-j)** A decomposition of isosurfaces shown in panel (a) into isosurfaces with **(g)** $B_z > 0$ (yellow), **(i)** $B_z < 0$ (blue), **(h)** the magnetic state in the tetrapod and **(j)** isosurface with $B_z = 0$ (dark blue).
