# Supplementary material for: Three-dimensional magnetic nanotextures with high-order vorticity in soft magnetic wireframes
Source: Nat Commun. 2024 Mar 11;15:2193. doi: 10.1038/s41467-024-46403-8 (PMC10928081; doi:10.1038/s41467-024-46403-8)
Supplement: Supplementary file 3 — Description of Additional Supplementary Files [file 41467_2024_46403_MOESM3_ESM.pdf]

Title: Supplemetary Video 1.
Description:This video visualizes the ansatz formula for the transformation between bulk and surface antivortices, which is presented in Supplementary Section 1 of the Supplementary Information.

Title: Supplemetary Video 2.
Description: This video shows the real-time energy minimization process for a tetrapod with a variable rotation angle. For information about the model and parameters, see Supplementary Section 2E of the Supplementary Information.
